# Annual Report of the Superintendents of the Poor of Erie Co.

**Published:** 1848-12

**Authors:** 


### EDITORIAL DEPARTMENT.

*Annual Report of the Superintendents of the Poor of Erie Co.*—From the official report of the superintendents of the poor of Erie County, as published in the Buffalo Commercial Advertiser, we derive the following statistics for the year ending Oct., 1848:—

| | |
|---|---|
| Whole number committed to the Erie County Poor House from Oct. 1, 1847, to Oct. 1, 1848, | 822 |
| On hand Oct. 1, 1847, | 162 |
| Whole number relieved, | 984 |

Of this number, 736 were paupers; 75 vagrants, and 11 lunatics. 189 children were sent to the Asylum, a branch of the Poor House, and contiguous to it. The number of lunatics on hand at the expiration of the year, amounted to 31; number of idiots 9; children 37; vagrants 10. Of the whole number committed during the year, and on hand Oct. 1847, 153 were native citizens, and 831 were foreigners. *Number of deaths at Asylum and Poor House during the year*, 159.

This enormous mortality is a melancholy commentary upon the *relief* afforded to the sick poor of the county of Erie, at the Poor House. We hope, for the honor of humanity, that a parallel cannot be found in the Poor annals of this country. According to the data above given, the ratio of deaths, in round numbers, is one to every six of the inmates of the Poor House, inclusive of vagrants, lunatics, children, and those paupers who are committed on account of destitution alone, as well as those committed on account of destitution and disease. One out of every six, (and a fraction) of them all, have died during the past year! It is with far other feelings than those of satisfaction that we call the attention of our readers to this statement. As a citizen of Erie County we do so with sorrow and shame. To obviate, however, any imputation of exaggeration in the tone of our remarks, we will cite the mortality in a few hospitals in different parts of the country, statistics of which are at hand.

At the *New Orleans Charity Hospital*, an institution which receives, gratituitously, all the sick who apply for relief, and which is supported by the state of Louisiana, the number admitted during the months of August, September, October and November, 1846, amounted to 3,571. The total of deaths, during the same period, amounted to 314. This makes a ratio of one to every eleven (and a fraction) of the whole number relieved. During the months given, it is stated in the report, contained in the New Orleans Medical and Surgical Journal, that Epidemic Dysentery was prevalent, and that many of the sick soldiers recently from Mexico were received. It will be observed that the mortality is nearly twice as large at the Erie County Alms House, as at the Charity Hospital at New Orleans!

By the Report of D. M. Reese, M. D., Physician to the *Bellevue Alms House Hospital,* we learn that, from May 25, to Aug, 1847, the whole number of patients received were 8,792. Of these 1,226 were cases of Typhus or ship fever. The number of deaths were 369; or one in every 23 (and a fraction). The mortality of the Erie Co. Alms House is thus nearly four times greater than at the Bellevue Alms House Hospital, near the city of New York! Dr. Reese's report is contained in the New York Journal of Medical and Collateral Sciences.

At the *Quarantine Hospital, Staten Island, N. Y.*, between June 6th and September 6th, 1847, there were received (as we gather from weekly reports published in the New York Annalist) 1,555 patients, the larger proportion being cases of Typhus, or ship fever. Of these 95 died. This makes a ratio of one to every sixteen and a fraction, a mortality almost one third less than at the Erie Co. Alms House!

We quote, lastly, statistics of *Grosse Isle,* where the mortality among the immigrants from ship fever was so great, that the place was characterized by the Montreal immigrant committee, in their report, as "the great charnel-house for victimized humanity." Between May 8th, and June 19th, 1847, the number of patients received at *Grosse Isle* was 2,796, four-fifths being cases of Typhus. The number of deaths was 565, or one in every 4-9 cases. Here the mortality exceeded that at the Erie Co. Alms House, but, as comparison of figures shows, by a small amount.

It is to be borne in mind, in the comparison with the institutions which have been named, that *they* are all devoted exclusively for the reception of the sick, while the Erie Co. Alms House receives vagrants, lunatics, idiots, and those who are merely destitute, but not sick. The report of the superintendents does not state how large a proportion of those committed were affected with disease, but making a very moderate allowance for the number not sick, and the ratio of mortality will exceed that of Grosse Isle. A fair comparison of mortality with other institutions like those named, it is obvious, requires that the ratio of deaths to the number actually under medical treatment should be ascertained. Throwing out one third of the whole number admitted (which is probably a small estimation) and the mortality is not only justly designated enormous, but, it is believed, to be without a parallel in any similar institution in the country.

Among the chief causes of this mortality, it is to be presumed, are the limited accomodations, and the want of appropriate nursing. The Poor House building was erected nearly twenty years ago, when Buffalo, compared with its present size, was but a small village. The only additions made since its erection, are, a small building for orphan children, small, one story wooden buildings for lunatics, and, during the past season, a temporary shanty for cases of Typhus, the cost of which (as stated in the report) was less than $500. That some idea may be formed of the capacity of the accomodations, in connection with the number of inmates, we give the following statement, which is quoted from a small tract entitled " An Appeal to the Citizens of Buffalo, and of the County of Erie, in behalf of New and More Efficient Means of Medical Relief for the Sick Poor," which was written and published seven years ago:—

"In the main building of the Alms House, are appropriated *two* wards for male, and *four* for female paupers, with a small bed-room in addition to the latter. The dimensions (length and breadth) of the male wards, are 16 by 28 feet Of the female wards, 15 by 20 feet; of the bed-room 6 by 15 feet.

The two male wards, at this moment, contain *twenty-four* persons.

The four female wards with the bed-room, *thirty-four* persons, four of whom are idiotic.

In addition to these, is a room over the wood-shed, 21 by 25 feet, with a contiguous chamber 12 by 21 feet. This is used as a work-shop and dormitory, and contains sixteen persons; two of whom are colored, and four idiotic.

All these wards fulfil the threefold functions of dormitory, sick ward and keeping room.

A small additional building was erected last autumn, which contains three rooms 17 feet 10 inches square. These are occupied by *twenty* parsons; three of whom are lunatics, one coloured, and three distinct families, i. e. men with wives.

This makes the whole number of inmates, at the time of writing, *ninety-four*. This is somewhat less than the average through the winter months. The number is, of course, constantly varying. At times it considerably exceeds *one hundred*.

In order to complete the representation, it is necessary to state, that there occur annually *ten* or *twelve* births in the house; that foundling and orphan infants are frequently brought there; and patients with every species, and in all stages of acute and chronic diseases; also, after accidents and injuries of every description."

Since the time the tract, from which the foregoing extract is taken, was published, the number of inmates has nearly doubled, and the ratio of mortality has increased (comparing the years 1842-3 with 1847-8) from one in every twenty, to one in about every six of the inmates. The mortality in 1842–3 was supposed, by the writer, at that time, to be very great, although more than one third less than during the past year.

During the five years of our medical service at the Institution, Typhus fever was once generated in the house, the cases of which were reported for the Boston Med. and Surg. Journal, June, 1841; at another season dysenteric-diarrhœa, of which there were fifty cases during the winter of 1842–3; and erysipelas during another winter. Since our connection with the Institution ceased, we have been informed that fever and erysipelas have been developed at different times, which the medical officers have not hesitated to attribute to concentrated animal exhalations, incident to over crowding. Now, it depends upon the Board of Supervisors to provide proper accommodations for the sick poor maintained at the expense of the county, and the subject has been repeatedly urged upon them. As County Physician, years ago, we made a report to the Board, stating the inadequacy of the accommodations, and that disease was actually generated in consequence; we stated, also, in that report, what we presume is still true, that patients not only died in the general wards surrounded by the sick, but corpses remained there (there being no vacant room for the reception of the dead) until taken away for interment, the moral effect of which upon the sick, and well, is readily conceivable; and, in a word, that, owing to defective accommodations and attendance, many died whose lives might, under more favorable circumstances, have been saved. The Board virtually acknowledged the justice of the claim upon them, on behalf of humanity, by passing a resolution appropriating a blank sum for the erection of suitable hospital buildings; but the sum was suffered to remain a blank! The subject has since been repeatedly urged upon their notice, but with no better success.

The great object with the Board of Supervisors, with respect to the poor, seems to be, to make the expenses for the support of pauperism as low as possible, and thus keep down the county tax for that object. Justice and humanity appear to be little thought of, in comparison with dollars and cents. The superintendents, and their subordinates, probably feel themselves under the necessity of acting in conformity with the predominant views of those from whom they received their places. May we not hope for better things now that the superintendents are responsible to the people directly, being elected by popular ballot? In making these remarks respecting the Board of Supervisors, we should state, that the majority of its members are from the country towns. Had the matter rested solely with the city representatives, who are so situated as to become more conversant with the merits of the subject, the evils of which we are speaking, would, we believe, long since have been remedied.

Next to want of accommodations, deficient nursing is involved in the the mortality. During the time we held the office of County Physician, we do not recollect that nurses for the sick were ever hired. This duty devolved upon fellow paupers, or vagrants, the latter being the most degraded persons that a city contains. How it has been of late we cannot speak from personal knowledge; but that very little has been disbursed for that object, may be surmised, when it is stated that the estimated cost of sustaining paupers at the Poor House during the last year was 59, 4-10 cents per week! not quite sixty cents a week for the support of an inmate of the Poor House! The Superintendents state this calculation with not a little complacency. They quote the cost of maintaining paupers in several other counties in which it doubles, trebles, and even quadruples the cost in the county of Erie—but they do not state in connection therewith, the comparison as respects the ratio of mortality! Indeed, the great mortality of our Poor House, in the report, is only noticed as adding about two cents per head to the cost of board per week! The matter does not seem (in the estimation of the superintendents) to call for any explanation, or comment, and, for aught the reader can gather from the report, the loss of life is as much a matter of congratulation with the superintendents and the Board of Supervisors, as the fact that they have cost the county less than 60 cents a head. We know not how it will strike others, but, in our view, the great economy which the report discloses, as compared with other counties, is no credit to us. Better far have footed up a larger amount of expenses, and a less number of deaths, If the citizens of Erie County are called upon to decide between life and money, we hope they have humanity and generosity enough to decide, not merely as tax payers, but as christian men.

But we have not yet presented all the statistics of mortality contained in the Report of the Superintendents of the Poor. The relief for the poor by the county embraces another arm than the Poor House. It has been, for many years, the policy in this county to expend a considerable sum for out door relief, i. e. to keep applicants out of the Poor House, (where was no room for them) by giving them a small weekly stipend. About twice the sum required for the Poor House was expended in this way during the past year. The operation of this system is to furnish a bounty for pauperism, and a premium upon deception—but we we do not purpose to comment upon it in those points of view at this time. *The uumber of deaths*

*in the city of Buffalo among those relieved out of the* **Poor** *House during the year, was* 290! Small as the chances of life have been at the Poor House, they have hardly been less, as the poor have been situated in the city. The Report does not state the number of persons relieved in the city of Buffalo during the year. It states only the number relieved throughout the entire county, which amounted to 5,509. A proportion of this number were relieved in the several towns of the county; 1,002, the Report states, were aided to get to their friends or places of destination. Now, deducting the latter, the ratio of mortality in the city of Buffalo, was one to every fifteen (and a fraction) of all assisted in the county! Of course a large proportion, it is to be presumed, were not sick, but only destitute, but the Report furnishes no data for determining the precise number of those needing medical aid, in addition to other assistance. Adding together the number of deaths at the Poor House, and among the paupers out of the Poor House, and we have a total of 449, which is not far from one third of all the deaths that have occurred in Buffalo during the past year! What the particular causes are which account for the great ratio of mortality among the out paupers, we will not now stop to consider.. But, we ask, can any reason be assigned why, if the sick, both in and out of the Poor House, had enjoyed the benefit of a commodious, and well regulated hospital, the mortality should have been greater here than at other places, for example, the city of New York? Is it not reasonable to assume that had the advantages for receiving and taking care of these patients been equal to those at Bellevue, the mortality would have been no greater? Observe, then, what would have been the difference in the result! at least *three-fourths* of the 449 that died during the year, would have been saved! Between three and four hundred lives (at the least calculation) would have been spared! Is this a matter of no interest, or importance in the minds of the citizens of Erie County? Is it a matter in which no moral responsibility is involved?

If this community fully appreciated the facts appertaining to this subject, there could not exist that apathy and apparent indifference toward it which now prevail. To suppose otherwise, would be to imply that the people of this County are not advanced in civilization so far beyond our predecessors, the Senecas, as we flatter ourselves we are. But, how is the subject to be brought to the apprehension of the public as it should be? We know of no way except through the efforts of the Medical Profession. It is peculiarly the province, and the duty, of medical men, to enlighten the public on subjects of this nature, and to incite to the full recognition of the claims of humanity toward the sick poor. Until this is done, in this County, the profession surely are not without an occasion for self-reproach, and they are, in some degree, involved in the responsibility which attaches to the present state of things. Let the practitioners of this city, and of the several county towns, set the matter in its proper light before the public mind, and they will have discharged the obligation which rests upon them, whatever may be the ultimate results of their exertions. The hope that our humble efforts may contribute, in some degree, to impress the force of this obligation, has prompted the foregoing remarks.

There are other matters contained in the Report of the Superintendents which admit of comment. We designed to have remarked on the number of lunatics, and the provisions for the proper management of mental maladies at the Poor House. It will have been observed that at the expiration of the year there were 31 lunatics on hand, and 9 idiots. The reader may well wonder whether justice can be done to this class of unfortunates, the number of which suffice for the occupancy of a tolerably large Lunatic Asylum. We quote the portion of the report which relates to this subject, in which it will be noted, that the chief point which appears to be regarded is the "*safe keeping aud confining of that unfortunate class of people.*" We must reserve discussion of this department of our system of *poor relief* for another number. The portion of the Report referred to, is appended as a postscript, to wit:—

"P. S. From necessity the Board of Superintendents feel called upon farther to submit to your honorable body more fully in this report, our present situation in reference to the Insane Department at the Poor House. We have thirty-four cells on hand in said department for the confining and safe keeping of that unfortunate class of people. While we have now on hand at the Poor House forty-one individuals of that class with whom there is no safety unless we have cells to confine them in—especially at night. Farther, there are three or four individuals now in the city of Buffalo, which are fit subjects of that department, and whom we necessarily shall have to take in charge immediately, which makes the number of eleven now virtually on hand, for which we have no place of confinement, for their own or others safety. In fact, in view of our present situation, and prospects ahead—judging from the past—we see no way but we shall be under the necessity of erecting some suitable building without delay for the reception and safe keeping of this class. And we would, therefore, recommend that such building be erected with capacity to accommodate at least twenty-five in number, additional to our present accommodations, which may require the sum of about one thousand dollars to complete the same. We would, therefore, recommend the raising of the sum of one thousand dollars for said purpose, in addition to the sum before asked for.

Respectfully,

J. S. KING,
LESTER BRACE,
*Superintendents of the Poor of Erie County, N. Y.*

October 13, 1848."

We should not do justice to the economy with which the medical department of Poor Relief is managed in this county, without noticing the salary which is allowed by the Superintendents for medical services. The Physician's salary for the past year (as we are informed) was $400, and the same salary is continued for the present year. For this sum it is expected that medical and surgical services will be rendered, not only at the Poor House, but to all the sick poor maintained by the County in the city, and medicines furnished!! It is, perhaps, a sufficient commentary on the emoluments of the office, to remark, that the compensation for medical attendance and medicines, is at the rate of less than a dollar a head for those who died during the last year! A salary of $400 for medical attendance and medicines for a number of cases of which 449 proved fatal!

But, to elucidate the matter of compensation more fully, we will consider it somewhat more minutely, with the aid of slate and pencil. In the absence of any precise statement with respect to the number of patients that came under medical treatment *in* and *out* of the Poor House, let it be assumed that they amounted to six times the number of fatal cases. To assume a larger number, would render still more striking the results at which we shall arrive; and to assume a smaller number, would involve a larger ratio of mortality than one in six. Upon the above assumption, there were treated, *in* and *out* of the Poor House, during the year, 2,694 patients. Medical and surgical attendance and medicines required for that number of patients were furnished for the sum of $400! According to this, the compensation for medical or surgical services rendered to each patient during the whole term of his illness, inclusive of all the medicines required, amounts to something less than 15 cents! The reader may now be curious to inquire respecting the average number of patients under treatment daily, and the daily compensation *per capitem.* In order to arrive at these points we must assume an average duration of illness for each case. Taking a low estimation, let it be *seven days.* Suppose, then, the average duration of each case to be seven days; the average number of new cases daily, according to the foregoing data, will be about seven. Hence, the average number of patients under daily treatment will be *forty-nine!* A portion of these cases, it will be borne in mind, were at the Poor House, and the remainder in different parts of the city. In order to form an idea of the relative number at the Poor House, and the number in the city, it seems fair to assume that the same relation which obtains between the deaths, will be applicable. Calculating from the relative numbers of deaths, there were 18 patients under daily treatment at the Poor House, and 31 to be visited, or prescribed for, in the city. Now the daily compensation, at a salary of $400 *per annum*, is a fraction over one dollar and nine cents, *per diem*. For about one dollar and nine cents *per diem*, then, 18 patients at the Poor House, and 31 in the city are expected to receive requisite daily medical and surgical attentions, inclusive of medicines! By a simple calculation it follows, that for medical or surgical services, inclusive of medicines, for each patient *per diem*, the County Physician received as compensation, the sum of a fraction over *two cents!* Curiosity suggests another arithmetical step, viz., to ascertain the average amount of the above sum which should be deducted for medicines, in order to reach the amount of *honorarium* which the county of Erie appropriates for professional services. Here, we confess, our arithmetic fails us. We must leave this interesting problem for some one more conversant, than ourselves, with the mysteries of the calculus; for, it is manifest, that it would lead us into infinitessimal quantities. The compensation which the present incumbent of the office of County Physician enjoys, is the same as that allowed for the last year, and, it is almost needless to add, the office for the last few years has not been held by the same person for two successive years.

